# Image Segmentation Based on Statistical Confidence Intervals

**DOI:** 10.3390/e20010046

**Published:** 2018-01-11

**Authors:** Pablo Buenestado, Leonardo Acho

**Affiliations:** Department of Mathematics, Universitat Politècnica de Catalunya-BarcelonaTech (EEBE), 08034 Barcelona, Spain

**Keywords:** image segmentation, statistical confidence interval, filtering, Otsu segmentation, speckle noise

## Abstract

Image segmentation is defined as a partition realized to an image into homogeneous regions to modify it into something that is more meaningful and softer to examine. Although several segmentation approaches have been proposed recently, in this paper, we develop a new image segmentation method based on the statistical confidence interval tool along with the well-known Otsu algorithm. According to our numerical experiments, our method has a dissimilar performance in comparison to the standard Otsu algorithm to specially process images with speckle noise perturbation. Actually, the effect of the speckle noise entropy is almost filtered out by our algorithm. Furthermore, our approach is validated by employing some image samples.

## 1. Introduction

Fundamentally, the image segmentation process is defined as the partition realized to an image into homogeneous regions [1,2,3,4,5]. The main objective of this process is to transform an image into something that is more meaningful and softer to examine by extracting important features from the original image information. Therefore, this subdivision will depend on the problem being solved. Nowadays, image segmentation is a fundamental stage in many engineering image preprocessing studies such as medical applications, video retrieval, pattern recognition, manufacturing inspection, structural fault diagnosis, et cetera [1,2,4,6,7,8,9,10,11,12].

On one hand, several segmentation approaches by using different concepts have been recently granted in the corresponding literature, just to name a few, see [4,5,8,9,13,14,15]. However, those techniques based on the thresholding design seem simpler [3,7,8,9,16]. Additionally, thresholding methods can be categorized into two main classes which are global and local thresholding, respectively. See, for instance [1,7,17,18]. In addition, there is a considerable number of thresholding algorithms, mainly based on histogram, spatial analysis, entropy, object attribute, and clustering [7]. Hence image thresholding is a well studied field. Among these is the well-known Otsu thresholding algorithm [8] which uses discriminant manipulation to find the maximum separability of classes [19]. Thus, its principal objective is to minimize the average error in classifying pixels among the group classes. Summarizing, threshold may be viewed as a statistical analysis for decision making. Furthermore, this operation is very simple by only utilizing the zeroth and the first order cumulative moments obtained from the histogram of the corresponding image under study. Lately, this design is straightforwardly extensible to the multivariate thresholding (or multiple thresholds) problems to extend it to an arbitrary number of classes to be identified in a reference image.

On the other hand, in statistics, a confidence interval is a kind of interval estimation of a population parameter that is computed from the experiment sample data. Hence, the confidence level is the proportion of a possible confidence interval that contains the true value of a corresponding probabilistic model parameter. It is important to highlight, however, that the interval computed from a particular sample does not necessarily include the true value of the estimated parameter. For instance, a 95 percent confidence interval reflects a significance level of 0.05. The most commonly level of confidence is set to 95 percent. In addition, a larger sample size normally will lead to a better estimation of the related population parameter. This theory is well documented in any scientific or engineering text book on probability and statistics, see for instance [20].

Lastly, for instance, the ultrasonic and SAR images have the main disadvantage to be contaminated by speckle noise [1,2,21,22]. The speckle noise is usually generated by the interaction of the reflected waves due to the interference of the returning wave at the transducer aperture. The existence of speckle noise significantly degrades the quality of an acquired image [21,22,23]. Thus, development of new techniques to face this external noise sensor perturbation is still an open research topic.

The novelty of this paper mainly lies in the evolution of a new image segmentation method by invoking the confidence interval theory and the well-known Otsu algorithm for segmentation. According to our numerical experiments, our method has a different performance in comparison to the standard based on the Otsu approach without using our pre-processing stage. This performance is specially notorious when our image is contaminated by the speckle noise. Furthermore, our approach is also validated by employing some image samples.

The rest of the paper is structured as follows. Section 2 presents our main contribution on image segmentation by using the previous cited statistical theory. Later, Section 3 gives the main theoretical discussion of our approach. The material and method are given in Section 4. Finally, Section 5 states the final conclusions.

## 2. Image Segmentation Based on Statistical Confidence Interval Approach

**Figure 1:**
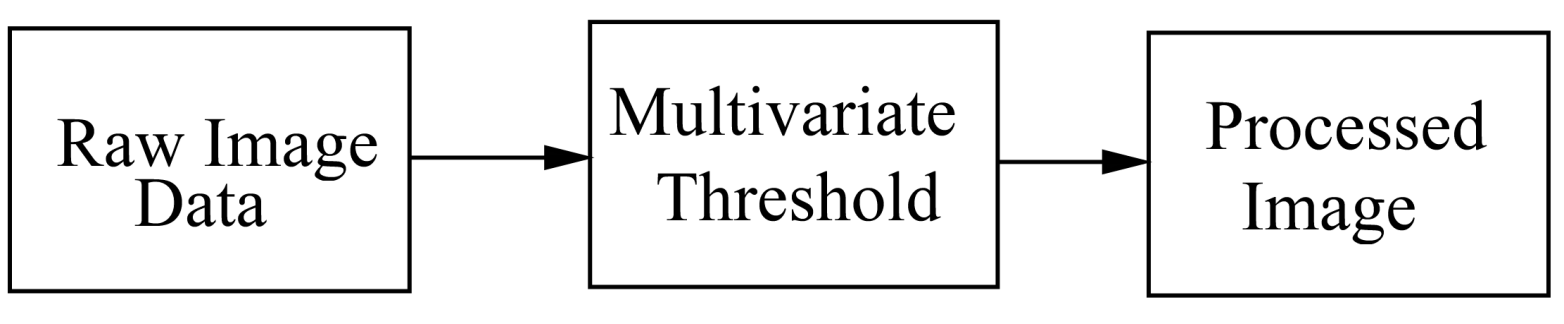
The standard image segmentation.

**Figure 2:**
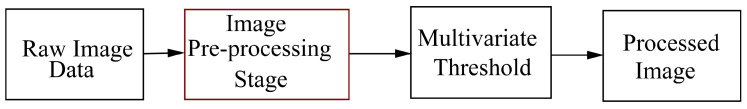
The proposed image segmentation.

**Figure 3:**
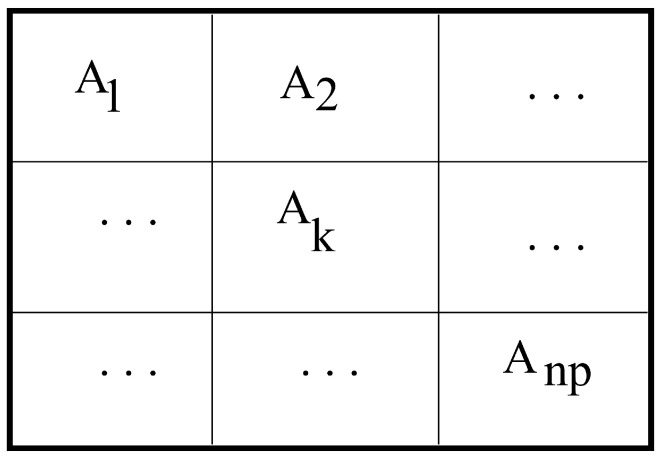
Sub-images representation.

In this section we present our main contribution. In order to compare our main part against a well-establish method, we present the following approaches. First, the standard image segmentation process is shown in Figure 1. It is important to note that noise perturbation affects the performance of image segmentation based on the Otsu method (see, for instance [7]). The shown multivariate threshold block is in fact the image segmentation stated by Otsu. The whole resultant process is here called the reference method. Second, our approach is shown in Figure 2. In this figure, in comparison to the previous one, we have added our pre-processing image stage technique. Once again, the multivariate threshold segmentation block is exactly the same as the standard method. This Otsu multivariate thresholding method is easily implemented by using, for example, the Matlab specially commands for image processing. In our numerical experiments, we use the three levels thresholding Otsu statement. Lately, our enhancement algorithm is set as follows (see Figure 2):

1. Obtain the standard deviation of the entire image population σ by considering each pixel intensity as a point-wise datum.
2. Separate the original image into $n_{p}$ sub-images. See Figure 3.
3. For each sub-image given by, for instance, $A_{k}\left(i,j\right)$ for $k=1,2,\ldots ,n_{p}$, where *i* and *j* is the Cartesian coordinate of a pixel and $A_{k}\left(i,j\right)$ is the related gray-scale pixel intensity, obtain its sample average value $\bar{x}$, and again, by considering each pixel intensity as a point-wise datum.
4. For each sub-image $A_{k}\left(i,j\right)$, for $k=1,2,\ldots ,n_{p}$; if $\left(A_{k}\left(i,j\right)<\bar{x}+1.96\sigma /\sqrt{i_{a}j_{a}}\right)$ and $\left(A_{k}\left(i,j\right)>\bar{x}-1.96\sigma /\sqrt{i_{a}j_{a}}\right)$, then $A_{k}\left(i,j\right)=A_{k}\left(i,j\right)$, otherwise $A_{k}\left(i,j\right)=\bar{x}$, for all *i* and *j* in the sub-image domain $\left[1\ldots i_{a}\right]\times \left[1\ldots j_{a}\right]$.
5. Reconstruct the Pre-processed image by compositing the above resultant sub-images.
6. Apply the above outcome image to the Otsu multivariate threshold process to obtain the final result. See Figure 2.

The above algorithm is really easy to implement by using, for instance, Matlab programming. In fact, we use it in our image numerical experiments. Then the final image segmentation was colored for gently human performance interpretation. On these experiments, we apply both our and the standard methods. Then, a discussion to the obtained results are given in the section that follows. Finally, we have assumed a confidence interval for the mean population, or expected value, μ, being a normal distribution with known $\sigma^{2}$ and a 95 percent confidence interval for it, that is:

(1) $$\bar{x}-1.96\sigma /\sqrt{i_{a}j_{a}}<\mu <\bar{x}+1.96\sigma /\sqrt{i_{a}j_{a}}.$$

**Figure 4:**
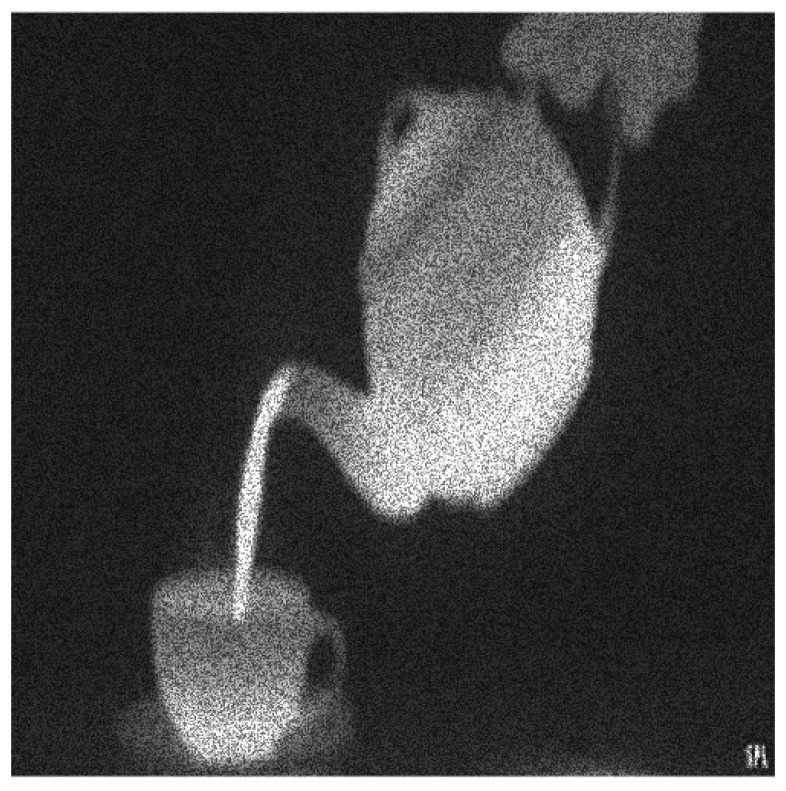
The original speckle noisy image (example 1) with σ=0.21714.

**Figure 5:**
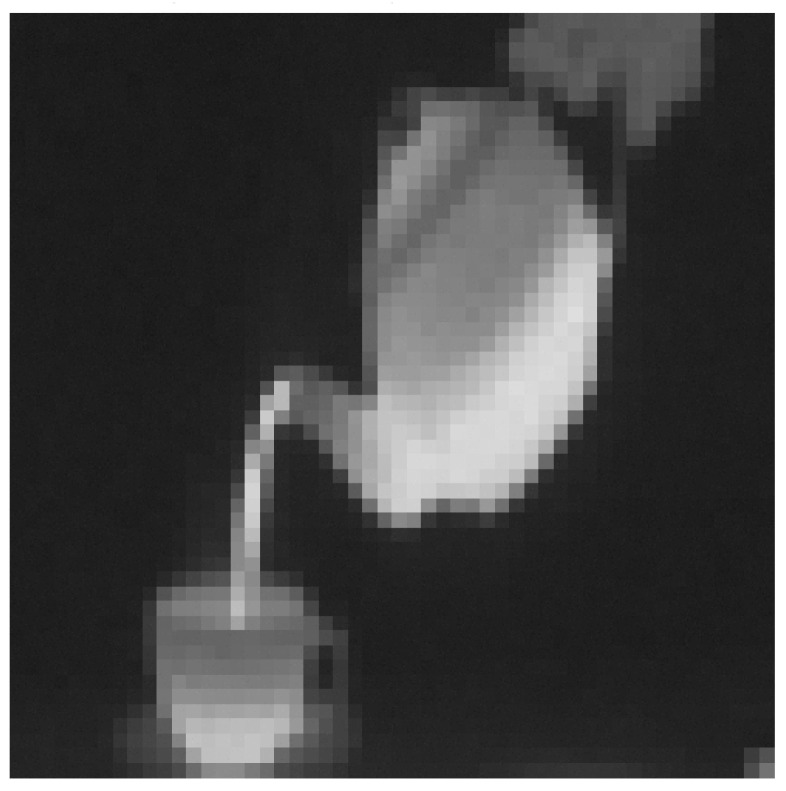
The pre-processed image by using our IC method (from the example 1).

**Figure 6:**
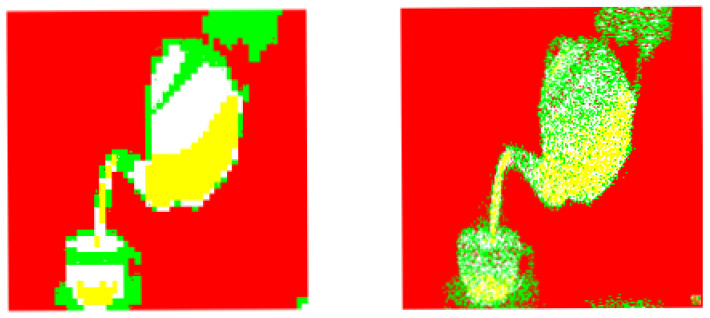
The corresponding image segmentations (from the example 1). Left: Processed image by using our design. Right: Processed image by invoking the reference method.

**Figure 7:**
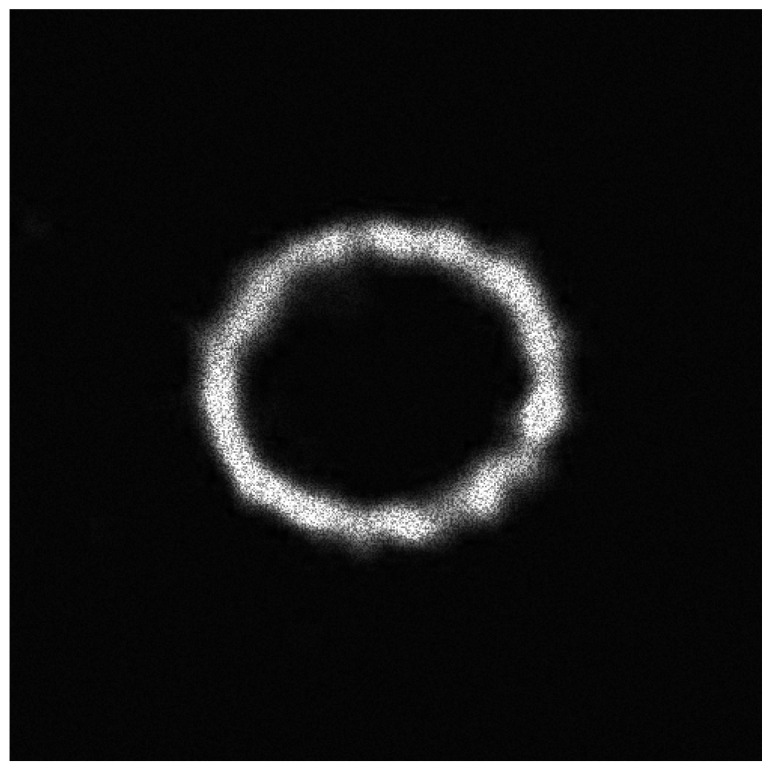
The original speckle noisy image (example 2) with σ=0.15854.

**Figure 8:**
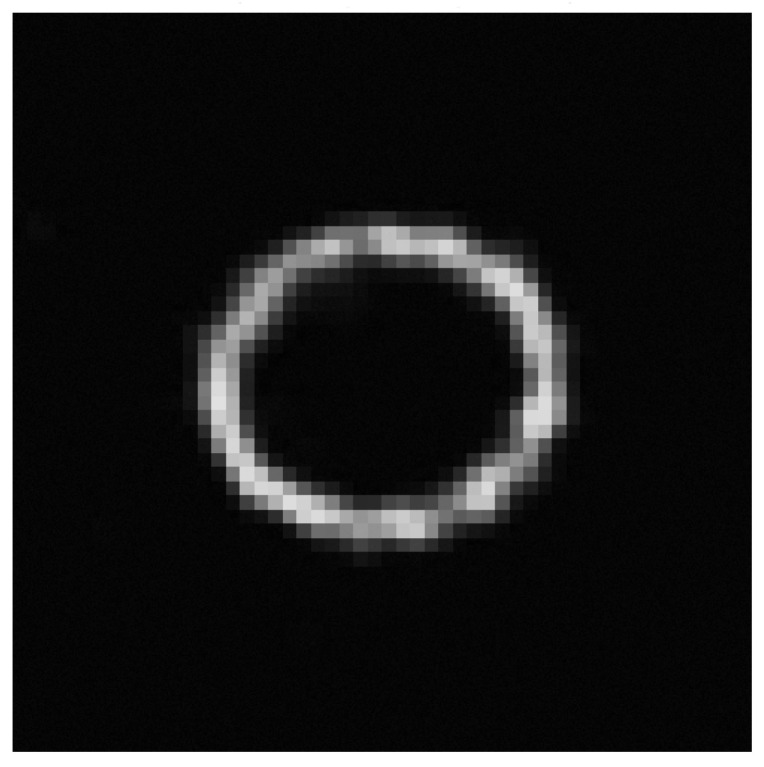
The pre-processed image by using our IC method (from the example 2).

**Figure 9:**
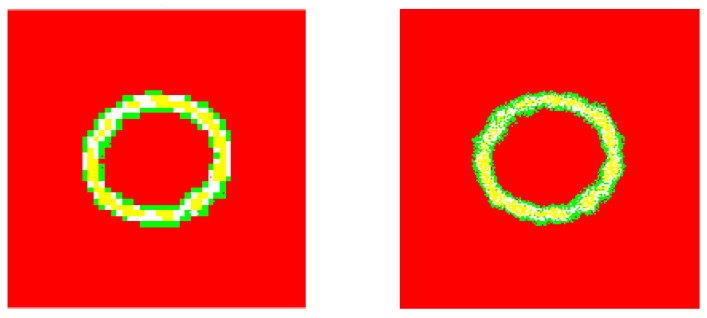
The corresponding image segmentations (from the example 2). Left: Processed image by using our design. Right: Processed image by invoking the reference method.

This late expression was employed in our computation algorithm. Obviously, we have implicitly assumed that our sample dataset size is large enough to validate the Central Limit Theorem. Some numerical experiments are shown in Figure 4, Figure 5, Figure 6, Figure 7, Figure 8 and Figure 9. Next are some remarks.

**Remark** **1.** Step 4 in our algorithm, we assign the value $\bar{x}$ to the corresponding variable in case when the conditional if is not satisfied. Obviously, other options can be realized. For instance, by using $max\{A_{k}\left(i,j\right)\}$ or $min\{A_{k}\left(i,j\right)\}$. However, we decide to use this average value $\bar{x}$ as an agent of the studied sub-image sample.

**Remark** **2.** Since a key assumption of Otsu’s method is based on the Gaussian distribution noise perturbation in our image, and because we are invoking statistical confidence interval theory, and according to the Central Limit Theorem, if $\bar{X}$ is the mean random variable of a given sample dataset of size n sufficiently large (usually n≥30), then the confidence interval analysis can be realized by employing the standard normal distribution representation even thought the speckle noise perturbation, or other, is in fact presented in our image dataset.

**Remark** **3.** In our numerical experiments, see Figure 4, Figure 5, Figure 6, Figure 7, Figure 8 and Figure 9, we use sub-image samples size of n=52 pixels to satisfy the Central Limit Theorem required to appeal the statical confidence interval approach given in Equation (1).

## 3. Discussion

In the previous section we have granted our recent algorithm on image segmentation based on confidence interval statistical theory. According to our numerical experiments, our method presents better performance in comparison to the standard process. For instance, from the first example, the processed image, in our case, was able to classify image segmentation more efficiently than the standard method. For the second example, it is clear that there is something around the object center almost imperceptible by the standard method. Finally, Equation (1) was invoked because according to the statistical theory, the confidence interval gives a zone where the population mean value is probably located. In our philosophy, this interval reflects the location of the hight content information versus the noise entropy.

**Figure 10:**
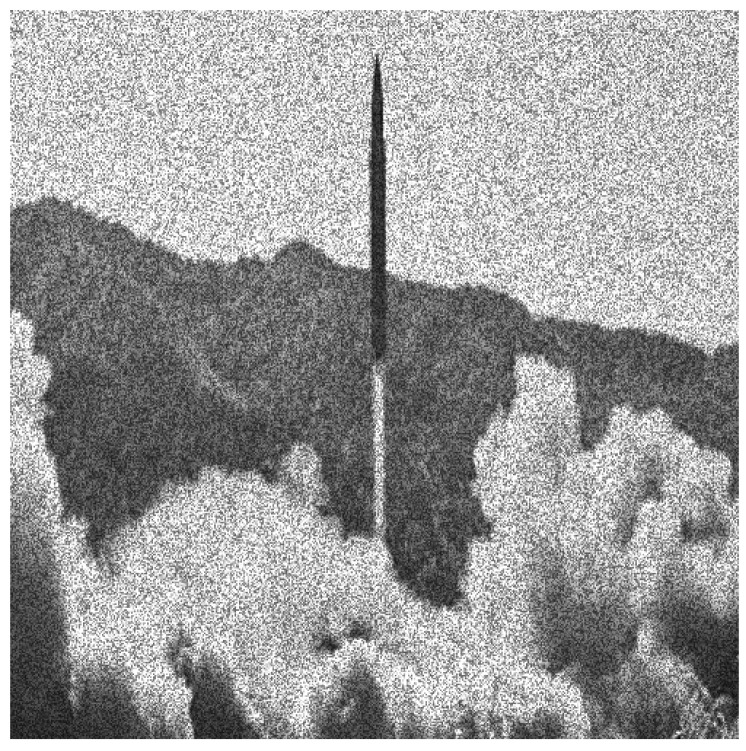
The original speckle noisy image with σ=0.27043.

**Figure 11:**
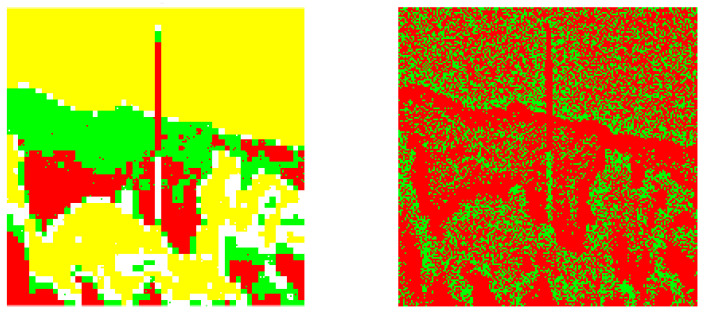
The corresponding image segmentations. Left: Processed image by using our design. Right: Processed image by invoking the reference method.

On the other hand, it is interesting to compare our approach to at least one other image segmentation technique. At this point, we program the binary segmentation process by using the Matlab adaptive image thresholding command adaptthresh with sensibility of 0.4 and then followed by the binary image segmentation process by invoking the Matlab statement imbinarize. By employing the speckle noisy image shown in Figure 10, the obtained result along with our is depicted in Figure 11. Even when the binary segmentation technique is employed for this alternative segmentation method, the presence of the speckle noise is still persistent. This validates the superiority of our approach.

**Figure 12:**
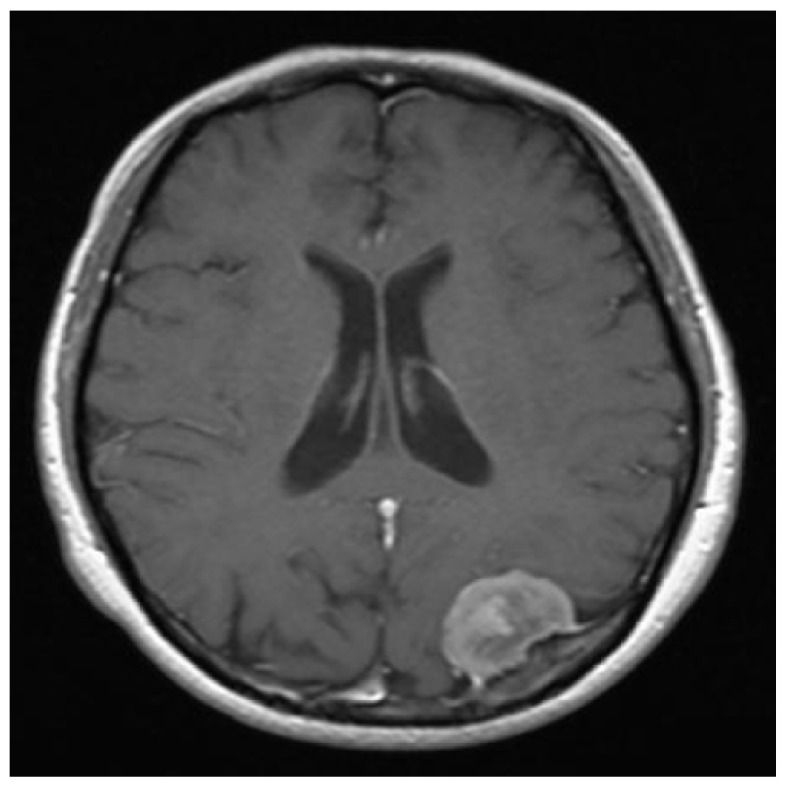
The original clean image.

**Figure 13:**
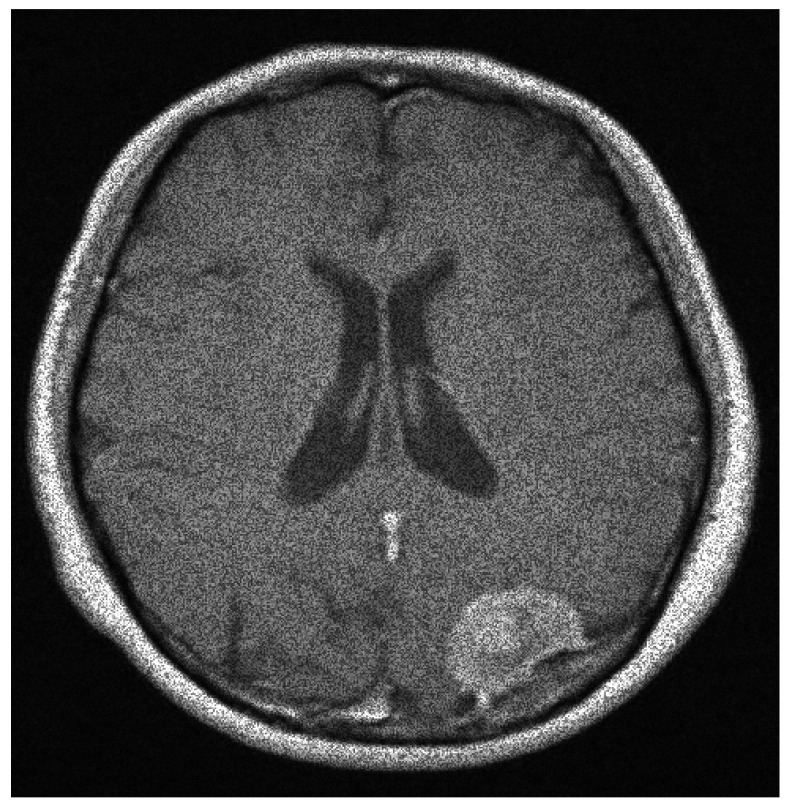
The speckle noisy image with σ=0.22152.

**Figure 14:**
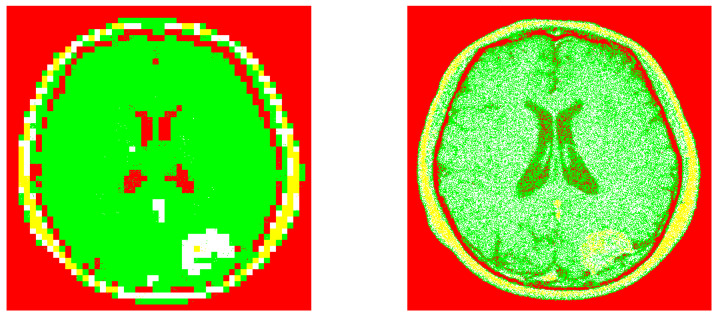
The corresponding image segmentations. Left: Processed image by using our design. Right: Processed image by invoking the reference method.

Moreover, and by accepting the fact that there is no still a satisfactory performance measure index for a image segmentation process [13]. So far, we utilize the subjective evaluation criterion [13]. However, it is important to note that even when this criterion is widely used, an untrained subjective evaluation may result in a mistake conclusion [13]. Then, by suing the subjective evaluation measure to our processed noisy images, our technique, and according to our numerical experiments, presents an acceptable performance. On the other hand, the Jaccard similarity coefficient has also been extensively used for the performance evaluation of image segmentation methods. Recall that the Dice similarity coefficient is related to the Jaccard one. Essentially, the Jaccard coefficient, also known as the Tanimoto coefficient, measures the overlapping of two given sets, or two images in logical format, where a value of zero indicates no overlapping, and a value of one indicates a perfect agreement. Hence, if two segmentation processes are going to be compared, the one with the bigger Jaccard coefficient (or closer to one) value will have the better performance. Therefore, we proceed as follows in order to validate our performance segmentation algorithm by employing this Jaccard similarity coefficient in comparison to the reference method. Given the original image shown in Figure 12, we add the speckle noise to it. See Figure 13. Then, by utilizing this noisy image, the processed segmentation results are depicted in Figure 14. Then we transform the clean image into a binary image (logical format) by invoking the command im2bw of Matlad with a thresholding level of 0.5. We called it Image A. After transforming the colored images shown in Figure 14 into gray-scale images, we also transform them to their binary formats by employing the same previous command at the same thresholding level too. We call them Image B and Image C, respectively. That is, Image B is the resultant binary image of our processed image, and the other one coming from the corresponding reference method. Now, we are interested in comparing the similarity between Image A and Image B with respect to the similarity between the Image A and the Image C by invoking the Jaccard method. By doing it, we obtain 0.6215 and 0.6215, respectively. Hence, our approach has a better performance.

**Figure 15:**
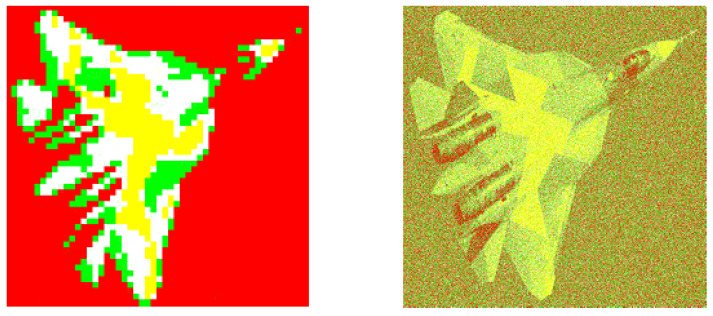
The corresponding image segmentations. Left: Processed image by using our design. Right: Processed image by invoking the reference method.

**Figure 16:**
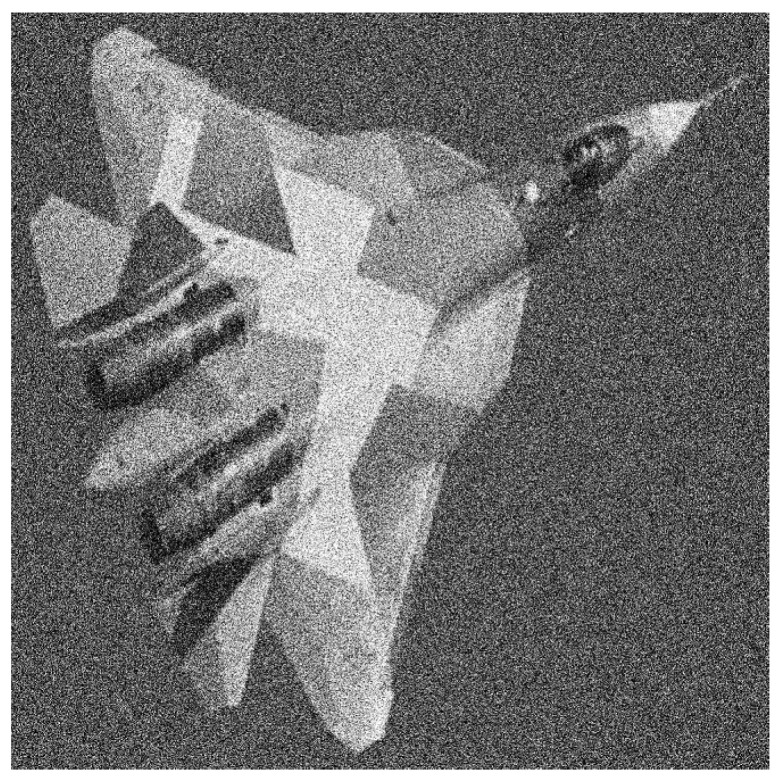
The original Gaussian noisy image. The Gaussian noise parameters are: zero mean and variance equal to 0.1 (σ=0.30964).

Additionally, and as was suggested by a reviewer remark, we realize an extra experiment but now by using Gaussian noise instead of the speckle one. The obtained results are shown in Figure 15 for the sample image given in Figure 16. Finally, and thanks to a reviewer observation, it is intriguing to observe that by binning and averaging an image into a sub-image samples, it results in obtaining a reduced resolution of the mentioned sub-image, and in consequence, of the whole image itself. Hence, this facilitates the segmentation image process. This is not totally true in our approach if we observe that our technique does not always produce this reduce resolution stage. See step 4 in our algorithm.

## 4. Materials and Methods

In this section, we present our algorithm realization approach on image segmentation by using Matlab programming (2017a, MathWorks, Natick, MA, USA). Basically, we employ this software platform to implement our procedure because it requires no programming experience, but some familiarity with it. In any case, Matlab is a powerful software package that has built-in functions to accomplish a diverse range of tasks, from mathematical operations to image processing. Additionally, Matlab has a complete set of programming routines that allows users to customize programs to the user own specifications. Furthermore, and because of we are dealing with image processing by following a well-established algorithm, the best way to describe the computational source material is to grant the programing lines employed to generate our numerical experiments. This is shown in the appendix.

The second part of Materials and Methods consists of appropriately selecting the test-images to appreciate our method on image segmentation. Principality, we are interested in images that have some salient visual property, especially for human visual perception. Therefore, we carefully selected images from different engineering fields: infrared, medical, military, and cosmic images.

Finally, we have the computational resource. Because we are employing the Matlab software with the corresponding tool box on image processing in it, we require a modern computer. We use a Windows 7 64 bits with Matlab version 2017a. Due to our images being small, the computation time is short. This gives us flexibility to run many numerical experiments.

## 5. Conclusions

In this paper we have proposed a new method on image segmentation by using a pre-enhancement stage based on a statistical confidence interval. According to our numerical speckle noisy image experiments, our approach has better performance than the standard Otsu method. Actually, the effect of the speckle noise entropy is almost filtered out by our algorithm. In our design, we implicit invoked the Central Limit Theorem requiring a sample size that is sufficiently large. However, if this sample size is small, instead, we can use the statistical confidence interval by utilizing the *t*-student probabilistic model. This may be interesting when contrasting both methods from their performance point of view. This is left as a future work. On the other hand, the Central Limit Theorem requires that the population probabilistic model has a mean and expected values. However, there are situations where this assumption is not satisfied, for instance, when our population model has the Cauchy distribution model (as was observed by a reviewer), where these values are undefined. Hence, applications where this distribution model is presented is also an open research topic. Moreover, and from the academic point of view, a programming platform competing against Matlab is Python. This because Python is a open source platform. Then, it is interesting to generate the given corresponding Matlab code by using this open source programming option. Additionally, in all our numerical experiments, we use grayscale images; obviously, the extension of our method to color images is also an open inquiry topic. Finally, while exploring the main property of the Jaccard coefficient as a performance index on image segmentation processes, and because we are using random data due the noisy perturbation, the results also propose a new performance index but from the statistical point of view.

## Appendix A. Matlab Programing Code

clear all;
%%%%%%%%%%%%%%%%%%%%%%%%%%%%%%%%%%%%%%%%%%%%%%%%%%%%%%%%%%%%%%%%%%%
%    Read the original image and add the simulated Speckle noise  %
%                and resizing it for simplicity                   %
%%%%%%%%%%%%%%%%%%%%%%%%%%%%%%%%%%%%%%%%%%%%%%%%%%%%%%%%%%%%%%%%%%%
AG1=double(rgb2gray(imread(’file_name’,’jpeg’)));
AGa=imresize(AG1,[1000 1000]);
AG=imnoise(mat2gray(AGa),’speckle’,0.1);
%%%%%%%%%%%%%%%%%%%%%%%%%%%%%%%%%%%%%%%%%%%%%%%%%%%%%%%%%%%%%%%%%%%
%  Obtain the corresponding standard deviation of the whole image  %
%           and show the noisy image to be processed              %
%%%%%%%%%%%%%%%%%%%%%%%%%%%%%%%%%%%%%%%%%%%%%%%%%%%%%%%%%%%%%%%%%%%
[I,J]=size(AG);
n=I*J;
x=zeros(n,1);
k=1;
for j=1:J
    for i=1:I
        x(k,1)=AG(i,j);
        k=k+1;
    end
end
s=std(x(:,1));
figure(1);imshow(AG);
title({[’Original Image’];[’\sigma= ’ num2str(s)]});
%%%%%%%%%%%%%%%%%%%%%%%%%%%%%%%%%%%%%%%%%%%%%%%%%%%%%%%%%%%%%%%%%%%
% Make the image partition into ’np(=52)’ sub-images and realize  %
%              the corresponding pre-processing steps             %
%%%%%%%%%%%%%%%%%%%%%%%%%%%%%%%%%%%%%%%%%%%%%%%%%%%%%%%%%%%%%%%%%%%
np=52;
nr=ceil(I/np)-1;
nc=ceil(J/np)-1;
 for n=1:np
     for m=1:np
         aux=((AG(1+nr*(n-1):nr*(n)+1,nc*(m-1)+1:nc*(m)+1)));
         xbar=mean(mean(aux));
         [ia,ja]=size(aux);
          for j=1:ja
              for i=1:ia
                  if (aux(i,j)<xbar+1.96*s/sqrt(ia*ja))&...
                  (aux(i,j)>xbar-1.96*s/sqrt(ia*ja))
                      aux(i,j)=aux(i,j);
                  else
                      aux(i,j)=xbar;
                  end
              end
          end
          A3(1+nr*(n-1):1+nr*(n),1+nc*(m-1):1+nc*(m))=aux;
     end
 end
%%%%%%%%%%%%%%%%%%%%%%%%%%%%%%%%%%%%%%%%%%%%%%%%%%%%%%%%%%%%%%%%%%%
%             Show the pre-processed image                        %
%%%%%%%%%%%%%%%%%%%%%%%%%%%%%%%%%%%%%%%%%%%%%%%%%%%%%%%%%%%%%%%%%%%
figure(4);
imshow(A3);title(’Pre-processed Image by using our IC method’);
%%%%%%%%%%%%%%%%%%%%%%%%%%%%%%%%%%%%%%%%%%%%%%%%%%%%%%%%%%%%%%%%%%%
%     Applied the corresponding Otsu segmentation method to       %
%      our pre-processed image and show the final result          %
%%%%%%%%%%%%%%%%%%%%%%%%%%%%%%%%%%%%%%%%%%%%%%%%%%%%%%%%%%%%%%%%%%%
level=multithresh(A3,3);
Aaux=imquantize(A3,level);
mymap = [1 0 0;0 1 0;1 1 1;1 1 0];
colormap(mymap);
rgbImage = ind2rgb((Aaux),mymap);
figure(7);subplot(1,2,1);
imshow(rgbImage);title(’Processed Image: Our Design’);
%%%%%%%%%%%%%%%%%%%%%%%%%%%%%%%%%%%%%%%%%%%%%%%%%%%%%%%%%%%%%%%%%%%
%     Applied the corresponding Otsu segmentation method to       %
%   the original image and show its result (for comparison)      %
%%%%%%%%%%%%%%%%%%%%%%%%%%%%%%%%%%%%%%%%%%%%%%%%%%%%%%%%%%%%%%%%%%%
level=multithresh(AG,3);
Aaux=imquantize(AG,level);
mymap = [1 0 0;0 1 0;1 1 1;1 1 0];
colormap(mymap);
rgbImage = ind2rgb((Aaux),mymap);
subplot(1,2,2);
imshow(rgbImage);title(’Processed Image: Ref. Method’);
%%%%%%%%%%%%%%%%%%%%%%%%%%%%%%%%%%%%%%%%%%%%%%%%%%%%%%%%%%%%%%%%%%%
%                          END                                    %
%%%%%%%%%%%%%%%%%%%%%%%%%%%%%%%%%%%%%%%%%%%%%%%%%%%%%%%%%%%%%%%%%%%
